# Promises of artificial intelligence in neuroradiology: a systematic technographic review

**DOI:** 10.1007/s00234-020-02424-w

**Published:** 2020-04-22

**Authors:** Allard W. Olthof, Peter M.A. van Ooijen, Mohammad H. Rezazade Mehrizi

**Affiliations:** 1 Department of Radiology, Treant Health Care Group, Dr. G.H. Amshoffweg 1, Hoogeveen, The Netherlands; 2 Department of Radiation Oncology, University of Groningen, University Medical Center Groningen, Hanzeplein 1, Groningen, The Netherlands; 3 Data Science Center in Health (DASH), Machine Learning Lab, University of Groningen, University Medical Center Groningen, Zielstraweg 2, Groningen, The Netherlands; 4 School of Business and Economics, Knowledge, Information and Innovation, KIN Center for Digital Innovation, Vrije Universiteit Amsterdam, De Boelelaan 1105, Amsterdam, The Netherlands

**Keywords:** Artificial intelligence (AI), Machine learning, Organizational innovation, Neurology/diagnostic imaging, Radiology, Technography

## Abstract

**Purpose:**

To conduct a systematic review of the possibilities of artificial intelligence (AI) in neuroradiology by performing an objective, systematic assessment of available applications. To analyse the potential impacts of AI applications on the work of neuroradiologists.

**Methods:**

We identified AI applications offered on the market during the period 2017–2019. We systematically collected and structured information in a relational database and coded for the characteristics of the applications, their functionalities for the radiology workflow and their potential impacts in terms of ‘supporting’, ‘extending’ and ‘replacing’ radiology tasks.

**Results:**

We identified 37 AI applications in the domain of neuroradiology from 27 vendors, together offering 111 functionalities. The majority of functionalities ‘support’ radiologists, especially for the detection and interpretation of image findings. The second-largest group of functionalities ‘extends’ the possibilities of radiologists by providing quantitative information about pathological findings. A small but noticeable portion of functionalities seek to ‘replace’ certain radiology tasks.

**Conclusion:**

Artificial intelligence in neuroradiology is not only in the stage of development and testing but also available for clinical practice. The majority of functionalities support radiologists or extend their tasks. None of the applications can replace the entire radiology profession, but a few applications can do so for a limited set of tasks. Scientific validation of the AI products is more limited than the regulatory approval.

## Introduction

Currently, artificial intelligence (AI) is a significant yet emerging technological innovation in healthcare. AI represents technologies that involve developing machines that can perform tasks that are characteristic of human intelligence [1]. Neuroradiology is one of the leading subspecialties in radiology in terms of the diversity and number of AI applications [2, 3]. Examples include the automated identification of stroke [4] and the automated volumetric measurement of multiple sclerosis lesions by artificial neural networks [5]. In this paper, we systematically assess the potential impacts of AI in neuroradiology and offer an overview of the state-of-the-art applications on the market.

Disruptive innovation has an impact on the tasks that professionals perform [6]. The term disruptive refers to a fundamental change in the way the work is traditionally conducted. In our study, we aim to assess how disruptive AI can be for the radiologist’s job: are radiologists replaced by AI, are the possibilities of the radiologist extended by AI or are the radiologists supported by AI?

Currently, AI, especially deep learning, is receiving much attention as a disruptive innovation in medicine, especially radiology. Based on the number of articles, we may wonder to what extent this is another case of temporary hype or if there are substantial clinical applications beyond the hype [7]. In addition to the high expectations regarding the impacts of AI on knowledge work [8], the fear of change and losing jobs is also salient [9].

Despite all the attention paid to AI in radiology, publications generally provide information on the technical aspects of AI or about its application in a specific domain to showcase examples of its potential impact on the job of the radiologist [2, 9, 10]. To what degree AI can support, extend or replace what radiologists used to do is not systematically analysed, especially through a comprehensive overview of the existing AI applications. It is essential to know the type of impacts that AI can bring about to better formulate reaction strategies for working with AI [11].

Despite its importance, systematic evaluations of the functionalities of AI applications that are offered to the market are scarce [10]. Thorough examination of the existing AI applications and their functionalities for the radiology workflow help us understand if and how AI will influence the daily practices of neuroradiologists. The assessment of the usage of technology and the impact of technology is a known scientific approach called technography [12].

Research on AI should include not only the development of algorithms but also the broader impact of AI, such as the impact on the daily work of the radiologist [13]. New technologies require new evaluation approaches [14], and a systematic technographic review could provide an objective counterbalance to personal, subjective opinions on the value of AI in clinical practice.

A technography follows the same approach as a systematic literature review. Instead of reviewing the publications, it reviews instances of ‘technological developments’ in a domain. Each record is evaluated in a predefined systematic way to provide an objective assessment of the current status of the technological developments, their characteristics, and their focuses. Technography enables us to map out technological developments and thereby identify gaps and suggest opportunities for future developments.

The radiology workflow contains many steps, and it is unknown whether current AI applications have an impact. To assess the impact of AI on the radiology profession, we need to scrutinize the functionalities of AI applications and map them to the radiology workflow. A clear overview of AI developments and detailed analysis of their functionalities help radiology departments and radiologists make more informed decisions and prepare for the future. It also helps researchers and application developers identify areas that are eligible for future development.

## Purpose

1. Obtain a comprehensive, systematic overview of AI functionalities for neuroradiology by performing an objective, systematic assessment of available AI applications.
2. Analyse the potential impacts of these applications on the work of neuroradiologists.

## Methods

### Study design

In this mixed-method study, the intersection of the promises of AI and the workflow of neuroradiology is explored. The factual information about AI companies and applications is combined with an assessment of workflow and their impacts based on qualitative codebooks. Our approach has some similarities with a PRISMA systematic literature review, but also some differences. The similarities are systematic storage of data, systematic analysis with a predefined codebook with definitions and clearly described reproducible methodology. The differences are that our sampling is not performed with a query in a literature database, the data is not extracted from scientific publications but from vendors’ websites and that the research question is not entirely predefined, but is exploratory, which required the collection of additional data during the research process (information about platforms, funding information, scientific validation information).

### Data collection and coding

**Fig. 1:**
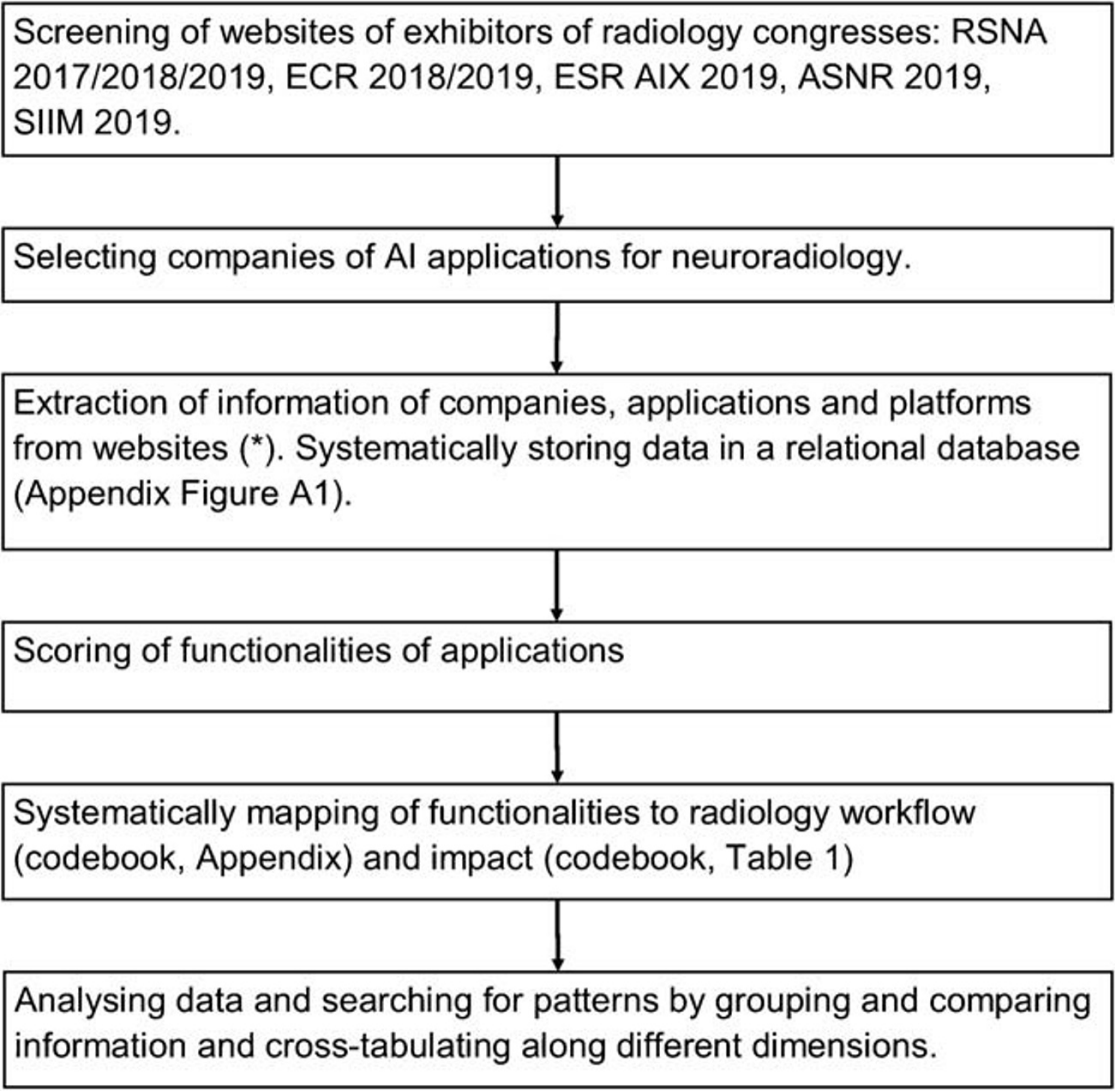
Research flow chart. RSNA = Radiological Society of North America. ECR = European Congress of Radiology. ESR AIX = European Society of Radiology Artificial Intelligence Exhibition. ASNR = American Society of Neuroradiology. SIIM = Society for Imaging Informatics in Medicine. Neuroradiology is defined as applications related to the brain or spinal cord as anatomical areas or related to specific diseases of the brain or spinal cord. (*) company websites, LinkedIn.com, Crunchbase.com, and the FDA website

Data collection, coding and analysis were performed, as indicated in Fig. 1. The conferences used as a source were selected based on their comprehensiveness or the subspecialties neuroradiology and imaging informatics. There was no restriction on the location of the companies. We did not consider non-commercial applications that also provide solutions, which are commonly used in research.

A radiologist with 10+ years of experience in neuroradiology (AO) performed the coding of the collected data by using codebooks to examine which tasks of radiology [15, 16] are targeted by an application and what kind of impacts it can have on these tasks (i.e. ‘supporting’, ‘extending’, ‘replacing’; Table 1). These tasks range from providing information to patients and referring physicians to the interpretation of an examination and the communication of results in a report or multidisciplinary team meeting [17].

**Table 1:** Codebook for classification of impact

Definition	Inclusion/exclusion criteria and examples
Supporting	
The functionality helps some inefficient task but does not fundamentally change the primary/current workflow, the interference of human actors is still required, and the process of task/workflow is still the same.	(1) does *not change* the entire task
	(2) make the process more efficient compared to prior activities
	(3) still *requires human involvement*
	(4) the system only helps humans to do their job
	Example: visualization the images and information
Replacing	
The functionality performs a certain task that was previously conducted by a human actor; thus, now the human actor is (almost) *not needed* for conducting this specific task.	(1) changes the particular fundamental task
	(2) does not require human involvement
	(3) technology *replacing the current human action*
	(4) the task was previously conducted by human actors
	Example: autonomous reading and reporting radiology cases
Extending	
Technology offers a functionality that was not previously performed by human actors or the previous systems, and now, with this new functionality, a *new task is added* to the work and may require the improvement of human capabilities.	(1) creates a *new task* in the workflow
	(2) does require human involvement but solves the problem through an algorithm
	(3) improves/extends *human skills and knowledge*
	(4) the tasks were previously non-existent
	Example: provide diagnostic information that was not available before, such as a heatmap of suspicious areas.

The reference point was the technological descriptions of the application and the use cases that are presented. For some functionalities, more than one of these impacts were selected (e.g. both supporting and replacing) when the impact could be multiple (e.g. depending on how the application is implemented in practice). The AI characteristics were scored based on a hierarchical classification [2], where deep learning is a subcategory of machine learning and machine learning is a subcategory of AI. If the vendor website did not offer a specification (machine learning or deep learning), we categorized the application under the broad category of ‘artificial intelligence’.

Applications without information on FDA approval were additionally checked at the public FDA website. For CE approval, no additional check was performed because there is no centralized public database.

### Database architecture

The primary data fields are ‘companies’, ‘platforms’, ‘applications’ and assessments of these applications. A relational database was designed to store and classify the data (Appendix Fig. 7 and Table 7). The data structure allowed one-to-many relationships: one company can have several applications, and one application can have several functionalities concerning various radiology tasks. Each application can have several types of approval certificates (e.g. FDA, CE marked), be used for several modalities (e.g. CT and MRI) and be related to different pathologies (e.g. dementia and stroke). Among the technological characteristics of the applications, information about PACS integration and whether the application works in the cloud or on-premise is collected.

In a separate list, we assessed the characteristics of the ‘platforms’ that we found during the search and analysis of AI applications. A platform in this context is the application through which the AI application is distributed and accessed (Table 2). Only platforms that are used for AI applications in neuroradiology are included. How an application addresses a platform is a distinctive feature and provides information on the usability of the application.

**Table 2:** Platform categories

Category	Explanation
Small/“Umbrella”	Applications of one vendor accessible through one umbrella product.
Intermediate/“Storage box”	Platform for in-house development of AI algorithms
Large/“Market place”	Platform of AI tools for developers and customers, similar to app-stores for smartphones.

## Results

### Overview of companies and applications

**Fig. 2:**
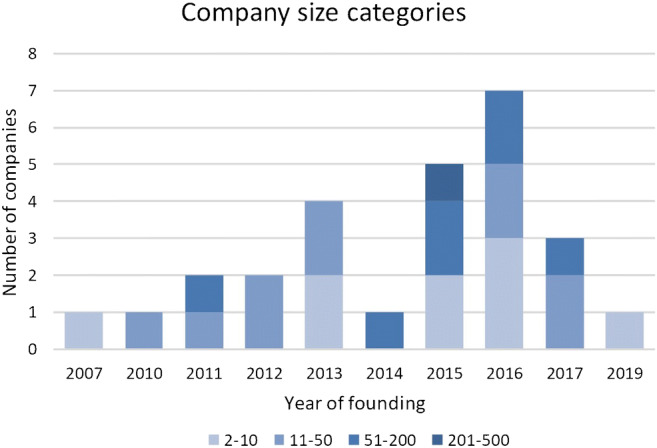
The founding year of the 27 companies and the distribution of the companies among the size categories, according to the number of employees

**Fig. 3:**
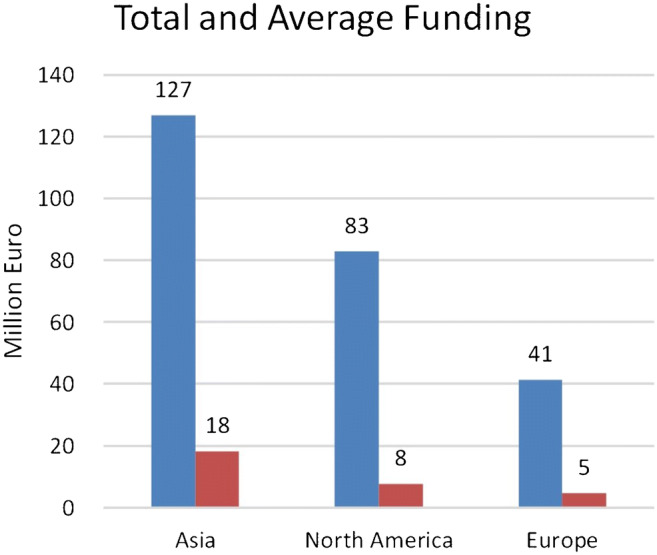
Geographical distribution of acquired funding of 13 of the 27 companies summed and averaged. The amounts in the national currencies have been converted to Euro. The average funding is calculated for all companies in each continent

We identified 37 applications of 27 companies from three continents. Most of the companies active in offering AI solutions are relatively small and young (Fig. 2). For half of the companies, we found information about their funding, which shows an uneven distribution of the amount of funding between the continents (Fig. 3). The majority of companies (74%) have a single product in this field (Table 3).

**Table 3:** Number of applications per company

Number of applications per company	Number of companies	Total number of applications
3	3 (11%)	9 (24%)
2	4 (15%)	8 (22%)
1	20 (74%)	20 (54%)

### Technological characteristics of platforms and applications

#### Platforms

We identified that applications could run on three types of platforms (Table 2). Some platforms are intended to be used with specific neuroradiology applications. Other platforms can be used to develop applications. The third group of platforms (‘marketplace’) provides many applications for many subspecialties within radiology (Table 4). Users can access the applications by using the platform.

**Table 4:** Application platforms

Description	Cloud_or_on-premise	Own_applications	Other_applications	Neuroradiology exclusive	Nr Neuro application^a^	Nr Application (total)^b^
Small/“Umbrella”						
ACCIPIO ICH Platform						
MaxQ AI’s diagnostic suite is being deployed directly onto both CT and PACS systems.	Both	Yes	No	Yes	3 (8%)	3
e-Stroke Suite						
e-Stroke Suite combines e-Aspects, e-CTA, and e-Mismatch.	Cloud-based	Yes	No	Yes	2 (5%)	3
Intermediate/“Storage box”						
CuraCloud						
AI Development Services supply medical imaging AI expertise and technical capabilities to healthcare organizations to create their own quality and productivity innovations using computer vision, machine learning, natural language processing, and other advanced informatics.	Cloud-based	Yes	Yes	No	1 (3%)	9
Incepto						
Incepto provides a collaborative environment to co-create, develop and distribute revolutionary applications for the diagnosis and treatment of diseases.	Cloud-based	No	Yes	No	1 (3%)	8
Large/“Marketplace”						
Blackford						
Blackford provides a single platform to access and manage a curated marketplace of regulatory approved medical image analysis applications and AI algorithms that add clinical value.	Cloud-based	Yes	Yes	No	4 (11%)	13
EnvoyAI						
EnvoyAI provides a developer platform, integrations and an API interface for algorithm developers, technology partners, and end users.	Both	Yes	Yes	No	11 (30%)	57
Nuance AI Marketplace						
Workflow-integrated market for diagnostic imaging AI algorithms.	Cloud-based	No	Yes	No	9 (24%)	25

^a^In brackets are the number of applications that indicate working with this particular platform. One application can be related to 1 or more platforms. For 19 (51%) applications, it is unknown whether they can work with a platform

^b^Total number of applications/tools available at this platform for both neuroradiology and other subspecialties

#### Applications

We categorized 12 (32%) applications under the broad category of AI, since they did not specify which type of algorithm they use. Machine learning (ML) was mentioned for eight (22%) and deep learning (DL) or convolutional neural networks for 17 (46%) applications. No specific information about technical details of algorithms or details about the training and validation data is available in the general information of the application websites.

Of all applications, 23 (62%) have seamless PACS integration, and 17 (46%) make use of cloud-based computing (Table 5).

**Table 5:** PACS integration and location of computation.

PACS-integration	
Seamless	23 (62%)
Manual	5 (14%)
Separate	1 (3%)
Modality integration^a^	3 (4%)
Unknown integration	7 (19%)
Cloud or on-premise	
Cloud-based computation	17 (46%)
On-premise computation^b^	5 (14%)
Location unknown	17 (46%)

^a^Three applications have modality integration, in addition to seamless integration

^b^One application is categorized as both cloud-based and on-premise computation

### Application regulatory approval

Thirteen (35%) applications have both FDA and CE approval, and 25 (68%) have at least one type of approval (Fig. 5). There is a regional variation in the percentage of applications with one or more approvals: Asia, 10 (91%); Europe, 8 (73%); and North America, 7 (64%). From the product of companies founded before 2014, 14 (88%) have one or more applications with one or more approval, compared with 11 (52%) of the applications that are offered by the companies founded in or after 2014.

### Modality and pathology type

All applications analyse images of one or more of the following imaging modalities: MRI (19; 51%), CT (19; 51%), MR perfusion (2; 5%), CT perfusion (3; 8%), CT angiography (5; 14%) and MR angiography (1; 3%).

Most applications are designed to be used for one pathology. The common pathologies are as follows: ischaemic stroke (13; 35%), intracranial haemorrhage (10; 27%) and mild cognitive impairment and dementia, including subtypes such as Alzheimer’s disease (7; 19%), multiple sclerosis (4; 11%), tumour (4; 11%), traumatic brain injury (3; 8%), Parkinson’s disease (2; 5%) and intracranial aneurysm (1; 3%).

In all three groups of regulatory approval (FDA, CE, other), the categories ischaemic stroke, intracranial haemorrhage and dementia are more frequent than the other categories are.

### Application functionalities and radiology workflow

Table 6 shows an overview of the functionalities of all applications, each accompanied by an explanation and an example. Figure 6 demonstrates the distribution of the functionalities over the workflow steps. One application can be mapped to one or more workflow steps.

**Table 6:** Functionalities with explanation and examples

Functionality	Count	Explanation and examples
Provides quantitative information about pathology	13 (12%)	Measures the characteristics of pathologic findings.
		Example: *InferRead CT Stroke* detects haemorrhagic stroke, marks the location and assesses the volume to assist radiologists in their diagnosis and determining the prognosis of a patient
Marks regions of interest or detects change	38 (34%)	Detects and highlights abnormal findings visually.
		Example: VIZ *LVO* uses artificial intelligence to automatically identify suspected large vessel occlusion strokes on CT angiogram imaging. *Change Detector* compares serial magnetic resonance imaging studies and presents changes in the form of a colour-coded change map.
Provides classification, diagnosis or outcome probabilities	19 (17%)	Interprets imaging findings and provides a diagnosis or a standardized classification.
		Examples: *Rapid Aspect* automatically generates a standardized score, based on clinically validated machine learning algorithms, that enables communication about the extent of a patient’s ischaemic changes. *Deepstroke* provides ASPECT scores and outcome probabilities based on different treatments.
Prepares report	15 (14%)	Organizes the diagnostic findings in a report.
		Example: *Atroscan* provides reports with quantitative information through comparative analysis of the same age group.
Automated derivation of brain biomarkers	12 (11%)	Compares the quantitative information about anatomy or pathology with normal findings of a particular group.
		Example: *Quantib ND* provides insight into the possible presence of atrophy related to Alzheimer’s disease or other types of dementia, thus supporting more accurate diagnosis, and makes use of reference centile curves of a population-based study.
Workflow optimization and triaging	12 (11%)	Facilitates the efficacy of the diagnostic process.
		Example: *RadReport* states that radiologists work faster and better with diagnostic decision support and standardized reports. *qER* includes a triage aid to prioritize and notify critical head CT scans.
Anatomical segmentation	2 (2%)	Segments the images in normal anatomical areas.
		Example: *Quibim Precision* is to designed to automatically calculate the volume of brain tissues and their regions and the mapping of local cortical thickness distribution.

### Impact on radiology work

**Fig. 4:**
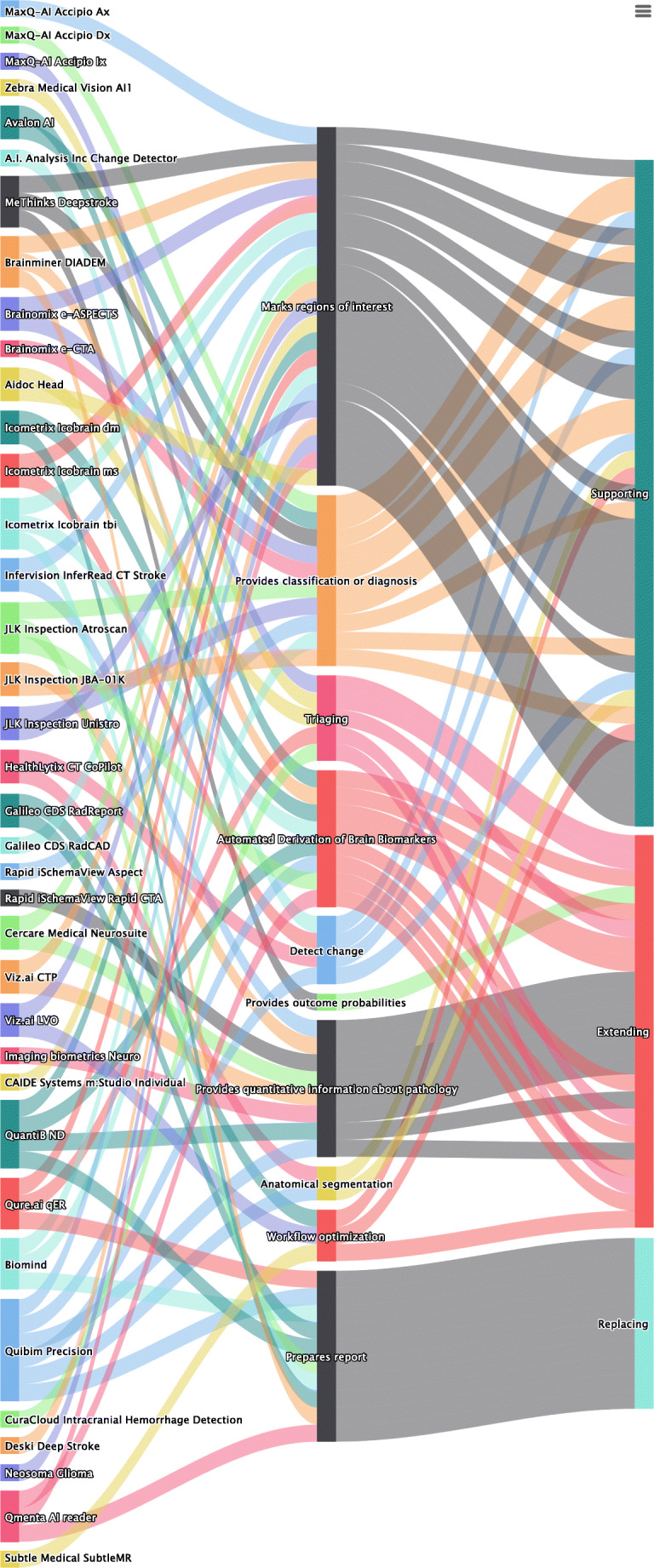
Sankey flow diagram. From left to right, the columns of items represent the companies, the functionalities and the impact. The size of each item corresponds to the relative value within the category. For example, the most frequent affordance is ‘quantification (pathology)’, and the most frequent impact is ‘supporting’

Most functionalities of applications (39; 54%) are designed to ‘support’ radiologists in performing their current tasks (Fig. 4). Some other functionalities of applications ‘extend’ the work of radiologists by providing quantitative information that would not be possible before the introduction of these applications (23; 32%). Only a few functionalities of applications (10; 14%) offer functionalities that take over certain tasks. A common example of replacing functionality is the preparation of a report. Both in the approved and not-yet-approved applications, the most frequent category is ‘supporting’, followed by ‘extending’ and ‘replacing’.

**Fig. 5:**
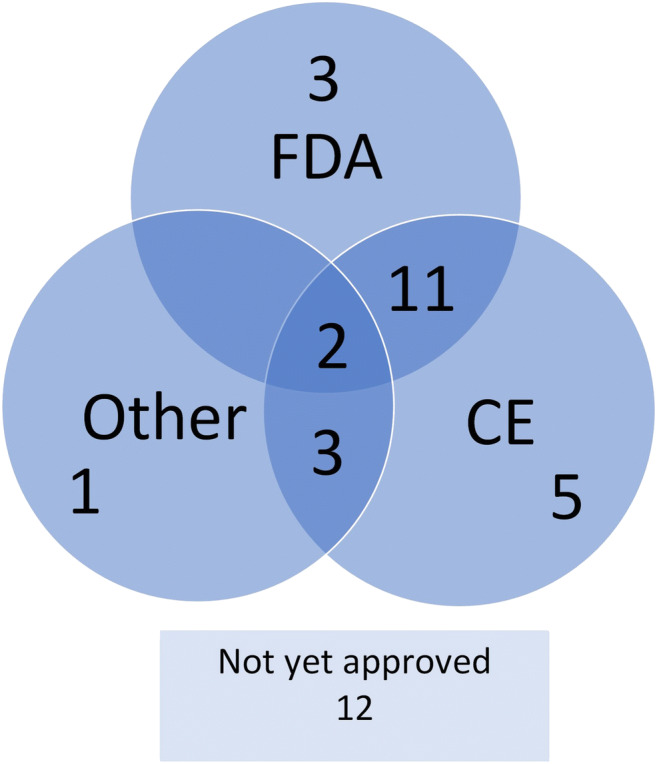
Approval of applications. Each circle represents several applications that are approved by a particular organization. The FDA is the Food and Drug Administration of the United States. CE means CE-marked. CE marking is a certification mark that indicates conformity with health, safety and environmental protection standards for applications sold within the European Economic Area (EEA). Numbers in the intersecting parts fall under two or more categories. The ‘other’ category represents the approval bodies of Australia, Canada, Korea, Singapore and Vietnam

**Fig. 6:**
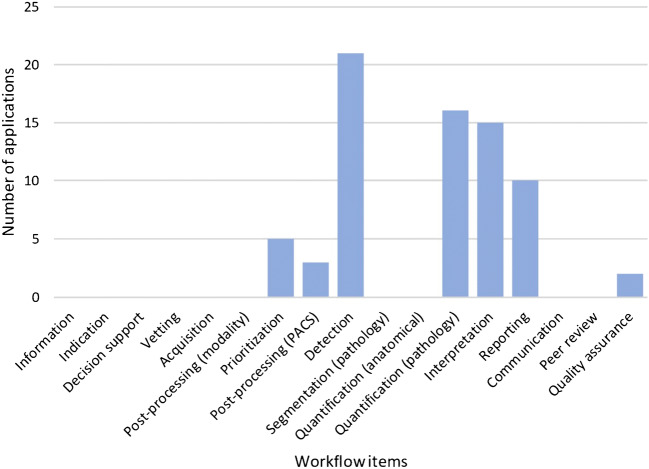
Workflow. The functionalities of each application are mapped to the items of the workflow of a radiologist, as described in the codebook in the methods section. Applications that detect and segment particular pathologic conditions are categorized under ‘detection’ and are not double-categorized under ‘segmentation (pathology)’. Applications that measure, for example, brain volume in the context of, for example, dementia, are categorized under ‘quantification (pathology)’ and not also under ‘quantification (anatomical)’

To illustrate the different categories, we provide several examples here. An application in the ‘supporting’ category is *e-Aspects* (Brainomix). This application automatically derives the aspect score for patients suffering from ischaemic stroke.

*Icobrain dm* (Icometrix) *extends* the possibilities of the radiologist by providing volumetric information about the brain and comparing this information with a normative database.

An example of an application that can *replace* the radiologist for a task is VIZ *LVO* (Viz.ai). The application alerts the on-call stroke team in case of large vessel occlusion.

An application sometimes offers functionalities related to all three categories. For instance, the *AI reader* (Qmenta) supports the radiologist in making a diagnosis, extends radiology work by providing quantitative information and can replace the radiologist in drafting an automated report for the referring physician.

### Scientific validation

Half of the applications offer some kind of scientific proof to show their validity. For five (14%) applications, the websites provide information about conference abstracts or publications, and for 13 (36%), there is both information at the websites and one or more peer-reviewed publications at PubMed. For 19 (51%) applications, there is no information about scientific validation.

## Discussion

This work describes a systematic technographic review of the functionalities and potential impact of AI applications and the characteristics of the vendors. Some vendors have more than one application, each for a specific task, while others have one application that can perform several tasks. Our approach addressed this by using a relational database [18] in which many functionalities or workflow items can be assigned to one application. This flexibility allowed us to conduct analysis not only at the application level but also at the functionality level.

### Companies and applications

The relatively high number of applications and the fact that most companies are young confirm the recent attention in the literature to AI in neuroradiology. Because of the limited information about funding, we cannot draw definite conclusions, but it is interesting to see that the companies leading the funding list are in China.

### Technical characteristics

For the majority of the applications, no information was available regarding the platform on which the applications run; thus, radiology departments will benefit from more detailed information from the vendors before they determine the application that best fits their needs.

For the majority of applications (68%), the type of AI (machine learning or deep learning) was provided. Some companies provide this information in detail, while others do not or provide it in a superficial manner. For radiologists, it is crucial to know the strength and weaknesses of the technology that they use to improve quality, ensure safety and understand artefacts [19, 20]. Additionally, radiologists need to understand technical information about the applications [21] to recognize the strengths and pitfalls of AI applications [22]. Information about the training data of algorithms and whether external validation was performed helps radiologists assess the credibility and applicability of an AI application in their hospital [23]. This type of data was limited. Close collaboration between radiologists and vendors is needed to ensure the true clinical utility of algorithms [24].

In addition to the algorithmic details of an AI application, the way the application can be integrated into the work environment has an impact on the job of the radiologist. Usability is essential to ensure that radiologists use the application in their daily work [25, 26]. The seamless PACS integration of many of the investigated AI applications facilitates radiologists’ efforts in using these applications. Even though our data do not show more detailed information about PACS integration, this finding indicates the awareness of vendors that integration in the daily workflow is essential for the adaption of the applications by radiologists.

### Regulatory approval

The fairly high percentage of approved applications demonstrates that AI in neuroradiology is not only in the state of developing and testing but also available for the radiologist in daily practice. Approval can be a starting point for evaluating the benefits of AI application for the health outcomes of patients, which requires higher levels of evidence than what is often needed for regulatory approval [27].

### Modalities and pathology

Neuroradiology heavily relies on MRI and CT, so it is not surprising that most applications are made to be used with MRI or CT data. The types of pathology that can be handled by the applications in our database reflect the frequently encountered diseases in neuroimaging. However, other major categories of disease for which neurologists and other specialists request imaging are missing, for example radiculopathy and epilepsy. In defining clinical challenges such as these, radiologists can contribute to translational research in artificial intelligence [28].

### Functionalities and workflow

The most numerous functionalities are directly related to the core business of a radiologist: finding and interpreting abnormalities and making the correct diagnosis. This fact indicates that AI companies develop products that are genuinely relevant to radiologists.

The items designated to the category ‘quality assurance’ are mainly designed to improve the workflow. No applications were found that perform a more direct quality assurance task, such as assessment of the completeness or quality of reports.

There is a shortage of applications and functionalities related to the early stages of the workflow (e.g. scheduling, acquisition and pre-processing) and the final stages (e.g. reporting and communication). This indicates the opportunities for companies and radiologists to develop applications in areas beyond image interpretation [29, 30].

### Impact on the job of a radiologist

Scientific journals dedicate papers and editorials to the emerging development of AI, wondering “Will Artificial Intelligence Replace Radiologists?” [31]. In general, AI will impact parts of many jobs, but other tasks within these same jobs will not change [32]. This is confirmed by our results. Currently, AI applications do not offer functionalities that can replace radiologists. The few applications that have the potential to replace the radiologist only can do that for a limited set of tasks, such as pre-drafting reports and analysing a stroke patient. In fact, the applications available on the market are still narrow-AI applications, meaning that they focus on one small task. This term can be applied to the AI tools that support or replace the radiologist for a single task, while the radiologist is needed to accomplish a sequence of other tasks. These applications do not check for other related or unrelated findings; therefore, the radiologist still has a task.

This fact does not mean that AI has no impact on the radiologist. Many applications are available, which can support radiologists, especially for the ‘detection’ and ‘interpretation’ of the clinical insights, the two primary responsibilities of a radiologist. Many applications also extend the work of radiologists. Quantitative information and biomarkers will enhance the content of the radiology reports of radiologists who choose to use these applications [33].

### Scientific validation

Companies are struggling with both scientific and regulatory validations of their products, though we see that the attempts to have sound scientific validation of the AI products are more limited than the regulatory approval. For only a minority of applications, peer-reviewed publications are available. This indicates that regulatory approval is not the same as clinical validation and confirms the remarks that most current applications are not yet ready to accept clinical deployment [34, 35]. The impact on patient outcome has yet to be assessed for almost all applications.

Reviewing new developments and providing an overview of the available applications is a well-established research approach. For example, Landau et al. provided an overview of AI in cytopathology and described both the literature and commercial landscape in a comprehensive review [36], Chen et al. described the current status of AI in urology [37] and Murray et al. performed a systematic literature review on AI applications in neuroradiology [38]. We found no other systematic technographic reviews similar to our study.

#### Generalizability

The applications that are designed for diseases such as stroke or dementia are specific to the neuroradiology subspecialty. These tools are not directly applicable to other subspecialties. However, the underlying concepts of the applications we investigated are generalizable to other types of pathology in neuroradiology or other subspecialties within radiology. These general concepts are as follows:

- the prioritization of studies in the PACS worklist, based on the presence of pathology
- the optimization of the workflow
- the quantification of anatomical structures and comparison with an age-based control group and the derivation of biomarkers
- the automated detection and segmentation of pathology
- the automated classification for pathology based on guidelines and specific criteria

This list indicates that radiology will not be the same in the near future. Substantial investments in AI will boost research and development in this domain [39].

#### Limitations

There is wide variation in the quality and completeness of the information on the websites of the vendors. Our results represent all available material that we thoroughly assessed. The characteristics of AI applications that are not publicly available were beyond the scope of our study, including applications that are commonly used in research institutions.

We included only applications that mention “neuroradiology” and “artificial intelligence” (or related words). However, some applications offer advanced AI tools for radiology that are also applicable in neuroradiology. Applications that use automated processing but that do not explicitly use AI were not included. Our results, therefore, might underestimate the applications that have an impact on the job of radiologists working in neuroradiology.

We included applications from exhibitors of several large radiology congresses in Europe and North America. Although several companies from Asia were present in our database, our results might be biased towards Europe and North America. Especially because of the high amount of funding acquired by some Asian companies, a significant contribution to future AI developments from this continent can be expected.

Another limitation is that we did not have interrogated the content of the scientific validation material. We only scored the presence or absence of this.

#### Future research

As mentioned, AI is developing at a high pace. Repeating our study over time helps us keep track of these developments and develop a more accurate overview of their potential impacts on radiology work and the radiology profession. This change over time will also provide valuable information about the development of this market. After-implementation feedback is also very important to determine how an application is actually used in terms of support, extension and replacement.

## Conclusion

Artificial intelligence in neuroradiology is not only in the stage of development and testing but also available for clinical practice. Many companies active in this area are young, ambitious and have acquired large amounts of funding. The applications developed are highly relevant for neuroradiology to support the radiologist and to extend the possibilities of the radiologist to add value to patient care. The main functionalities that support radiologists are the detection and interpretation of abnormal image findings. The primary functionality that extends the possibilities of radiologists is the provision of quantitative information. In the category ‘replacing’, some applications are available that make radiology reports in specific domains. Scientific validation of the AI products is more limited than the regulatory approval.

## Figures and Tables

**Fig. 7:**
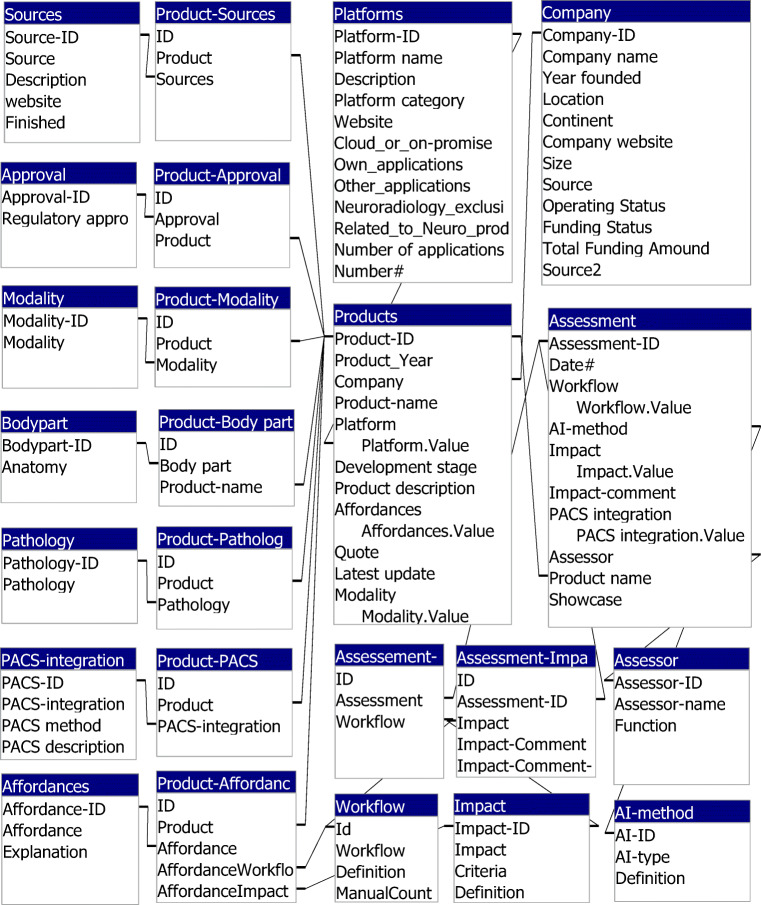
Database structure. For data storage, a relational database was developed in Microsoft Access. For analysis, information from different tables can be combined in queries and exported for visualization

## Appendix

**Table 7:** Workflow codebook

Workflow	Definition
Information	Provide information to patients and referring physician about a radiological examination
Indication	Decide the indications for certain examinations, for example implementation of institutional guidelines that describe the indications for specific radiologic examinations
Decision support	Support referring physician in choosing an examination for a specific patient
Vetting	Decide what imaging protocol is needed for a specific patient
Acquisition	Give input to technicians during image acquisition about adaptations to the imaging protocol in case of unexpected findings or other questions of the technicians.
Post-processing (modality)	Perform post-processing, before sending images to PACS
Prioritization	Decide the order in which images are read by the radiologist
Post-processing (PACS)	Perform post-processing steps during case reading, including anatomical segmentation
Detection	Detect and annotate abnormal findings
Segmentation (pathology)	Segment abnormal findings
Quantification (anatomical)	Quantify certain anatomical structures
Quantification (pathology)	Quantify abnormal findings
Interpretation	Interpret the detected normal or abnormal imaging findings in the context of the clinical history and the request of the referring physician; the cognitive process of going from imaging findings to a differential diagnosis.
Reporting	Report in terms of free-text or structured reporting
Communication	Communicate radiological findings by other means than the radiology report, for example, in a Multidisciplinary Team Meeting or by phone in case of the communication of critical findings.
Peer review	Analyse cases for peer review and provide feedback to other radiologists
Quality assurance	Perform tasks related to quality assurance such as improving of the workflow, or assessing the quality of radiology reports
